# Unusual presentation of sporadic Burkitt’s lymphoma originating from the nasal septum: a case report

**DOI:** 10.1186/1752-1947-7-60

**Published:** 2013-03-08

**Authors:** Gaffar Aslan

**Affiliations:** 1 Department of Otorhinolaryngology-Head and Neck Surgery, Istanbul Bilim University Faculty of Medicine, Istanbul, Turkey

## Abstract

**Introduction:**

Burkitt’s lymphoma is a highly aggressive, small, non-cleaved B-cell non-Hodgkin's lymphoma. In the sporadic form of the disease that occurs in non-endemic areas around the world, most commonly in developed countries, patients usually present with an abdominal mass that frequently involves the ileocecal region of the bowel; ocular or orbital involvement is rare. Primary disease of the sinuses is uncommon and, to the best of our knowledge, that of the anterior septum has never been described. We report the diagnosis and successful management of Burkitt’s lymphoma originating from the nasal septum in a male patient.

**Case presentation:**

An otherwise healthy 78-year-old Caucasian man who did not smoke cigarettes was admitted to our Ear, Nose and Throat outpatient clinic with the complaint of nasal obstruction due to left-sided nasal septal thickening. Paranasal computerized tomography revealed a well-circumscribed solid mass originating from his anterior nasal septum and obstructing his airway. The final diagnosis of Burkitt’s lymphoma was verified by immunohistochemical studies. Our patient had a good clinical outcome after chemoradiotherapy, with no problems reported to date in the second year of follow-up.

**Conclusion:**

We provide what we believe to be the first report of a case of sporadic Burkitt’s lymphoma involving the nasal septum, and describe the efficacy of first-line chemotherapy. Being an original case report with broader clinical impact across more than one area of medicine, this case presentation has the potential to significantly advance our understanding of Burkitt’s lymphoma and we emphasize the need to include this disease in the differential diagnosis of patients presenting with a nasal septal mass.

## Introduction

Burkitt’s lymphoma (BL) is a highly aggressive, small, non-cleaved B-cell non-Hodgkin's lymphoma that was first described in 1958 as a mandibular malignancy in African children and classified as a mature B-cell neoplasm under the World Health Organization classifications [1,2]. BL is endemic in certain regions of equatorial Africa, where it commonly involves facial bones, such as the mandible, maxilla and orbit, and can invade adjacent orbital soft tissue structures, such as the eye [3,4]. In the sporadic form of the disease that occurs in non-endemic areas around the world, most commonly in developed countries, patients usually present with an abdominal mass that frequently involves the ileocecal region of the bowel [5]; ocular or orbital involvement is rare [6]. Primary disease of the sinuses is uncommon and, to the best of our knowledge, that of the anterior septum has never been described. We describe the case of a patient with sporadic BL originating from his nasal septum, leading to the complaint of nasal obstruction due to left-sided nasal septal thickening, who responded well to chemotherapy.

## Case presentation

**Figure 1:**
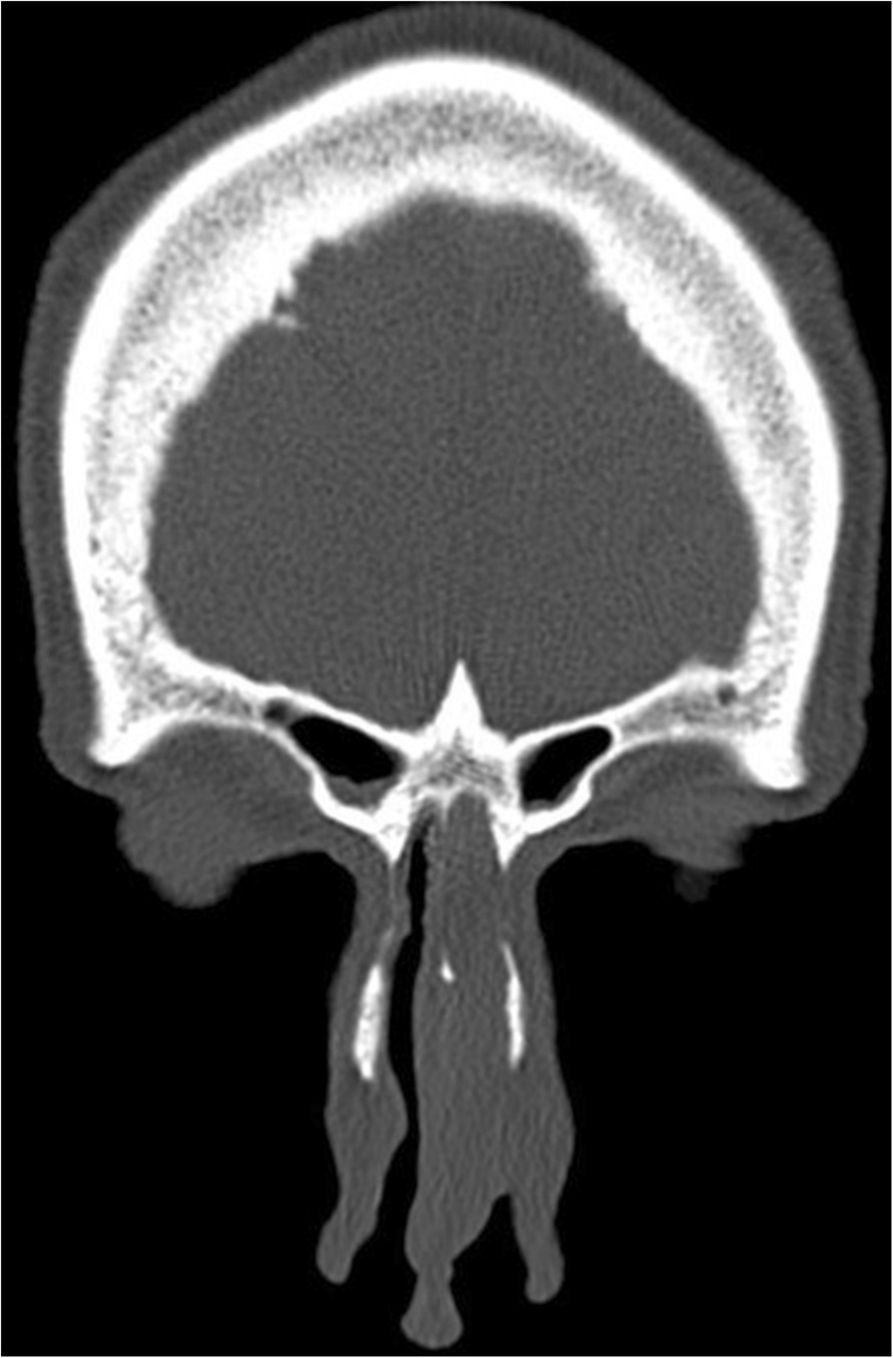
Coronal view on computed tomography showing a well-circumscribed septal mass.

**Figure 2:**
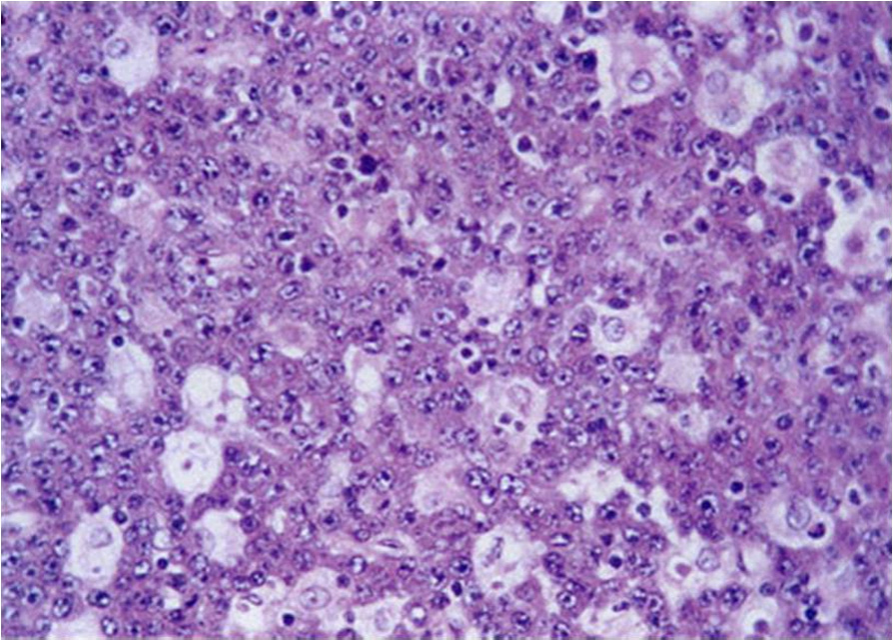
Histopathology demonstrated medium to large atypical lymphocytes and a ‘starry sky’ pattern of small, undifferentiated, tightly packed lymphocytes interspersed with large pale histiocytes (hemotoxylin-eosin, ×400).

An otherwise healthy 78-year-old Caucasian male patient who did not smoke cigarettes presented to our Ear, Nose and Throat outpatient clinic with the complaint of nasal obstruction. A physical examination revealed a smooth surfaced mass occluding his left nasal orifice with rightward deviation of his nasal septum. Paranasal computerized tomography (CT) revealed a well-circumscribed solid mass originating from his anterior nasal septum and obstructing his airway (Figure 1). A histopathologic evaluation of a specimen obtained via an incisional punch biopsy identified mucosal infiltration by lymphoid cells with small round nuclei and nucleoli and a ‘starry sky’ pattern of small, undifferentiated, tightly packed lymphocytes interspersed with large pale histiocytes (Figure 2). Following oncology consultation, a final diagnosis of stage 3 (Ann Arbor Staging System) BL was made and verified by immunohistochemical studies. BL, regardless of subtype, typically expresses monotypic surface immunoglobulin M and pan-B-cell antigens, including cluster of differentiation (CD)19, CD20, CD22 and CD79a, and co-expresses CD10, B-cell lymphoma 6 protein, CD43 and protein 53, but not CD5, CD23, B-cell lymphoma 2 protein, CD138 or terminal deoxynucleotidyl transferase. A test for human immunodeficiency virus revealed that our patient was seronegative for the virus; a titer for Epstein-Barr virus (EBV) was also negative and his lactate dehydrogenase level was elevated.

Eight scheduled cycles of chemotherapy as a first-line treatment with a combination of doxorubicin, vincristine, cyclophosphamide and prednisone were administered. Our patient received no radiotherapy sessions because control positron emission tomography imaging performed after the chemotherapy revealed no pathological findings. Post-treatment controls revealed a loss of overall tumor mass with no obstruction in both his nasal orifices. Due to identification of the well-circumscribed anterior mass, the control CT was not considered necessary. Our patient had a good clinical outcome after the chemotherapy and has reported no problems to date in the second year of follow-up.

## Discussion

Currently classified as a mature B-cell neoplasm under the World Health Organization classifications, BL was first described as a mandibular malignancy and documented in sub-Saharan Africa in a pediatric population [1,2]. It is now recognized to occur in three major variants. The endemic (African) form occurs in regions of equatorial Africa, in association with EBV infection in 95% of cases, and commonly involves facial bones [3,4]. The immunodeficiency-associated form occurs as a manifestation of human immunodeficiency virus infection and acquired immunodeficiency syndrome [7,8]. Finally, the sporadic (North American) form occurs in non-endemic areas around the world, where it typically manifests as an abdominal tumor [9]. Indeed, this small, non-cleaved B-cell lymphoma accounts for between 40% and 50% of childhood non-Hodgkin’s lymphomas in non-endemic areas [10,11], whereas the sporadic form in adults accounts for 1% to 2% of all adult lymphomas in western Europe and the United States [12].

Although BLs occurring in non-endemic areas are identical to those in endemic areas on histology, BL will often present differently depending upon the variant [12]. In this respect, our findings of typical histological features but an unusual presentation of sporadic BL, originating from the anterior nasal septum in a 78-year-old male patient, support that BL, as a mature B-cell non-Hodgkin's lymphoma, is a monoclonal proliferation of B-lymphocytes that can arise from any location in the body and demonstrates the ‘starry sky’ pattern of lymphocytes on histologic examination [6].

To the best of our knowledge, our case seems to be the first report of a nasal septal origination of sporadic BL in adults, as the lymphoma tends to arise in the lymphoid tissues of the gut and the upper respiratory tract, often presenting as masses in the Waldeyer ring or the terminal ileum, or even with massive abdominal involvement [12].

Given that primary sinonasal non-Hodgkin's lymphomas usually manifest with symptoms mimicking chronic sinusitis, including nasal obstruction, headache and post-nasal drip [13-15], the diagnostic work up should include appropriate laboratory investigation for differential diagnosis, and detailed CT imaging for an accurate identification of the location and extent of the tumoral mass to guide a biopsy. The most important diagnostic criteria is tissue diagnosis, after which all these investigations could be performed.

Although treatment is planned according to histopathologic subgroups, chemotherapy with a combination of cyclophosphamide, vincristine, methotrexate and prednol has been considered as the preferred therapeutic option in BL regardless of the stage of the disease. The efficacy of high dose cyclophosphamide, which achieves a treatment response in 80% to 95% of patients with endemic BL, has led to it being considered the cornerstone of treatment, either as a monotherapy or in combination with other chemotherapeutic agents [9]. Radiotherapy is considered to be not effective *per se*, but it can be added to the treatment plan according to the location of the tumor [16].

The prognosis for many patients with BL has changed significantly with the introduction of short, intensive chemotherapeutic regimens that have excellent response rates, with a 92% two-year event-free survival for children and adults with small non-cleaved lymphoma [12]. The selection of first-line chemotherapy with a combination of doxorubicin, vincristine, cyclophosphamide and prednisone followed by the radiotherapy in our case resulted in the complete cure of our patient with no problems encountered within the two-year follow-up.

In addition, given that the relationship of sporadic BL and EBV is controversial, with an indication that patients with EBV antibody positivity have better outcomes [17,18], it is of note that our patient had negative EBV titers which seems to be associated with a good prognostic outcome.

## Conclusion

We provide what we believe to be the first report of a case of sporadic BL involving the nasal septum. Our findings emphasize that, in patients presenting with a nasal septal mass, BL should be considered in the differential diagnosis and that a punch biopsy rather than total septal excisional biopsies should be performed in the case of septal tumors. First-line chemotherapy seems to be an effective therapeutic option in BL, and avoids permanent septal perforation and related unnecessary surgical approaches. Being an original case report with broader clinical impact across more than one area of medicine, this case presentation has the potential to significantly advance our understanding of BL in terms of the differential diagnosis of patients presenting with a nasal septal mass.

## Consent

Written informed consent was obtained from the patient for publication of this case report and accompanying images. A copy of the written consent is available for review by the Editor-in-Chief of this journal.

## Abbreviations

BL: Burkitt’s lymphoma; CD: Cluster of differentiation; CT: Computerized tomography; EBV: Epstein-Barr virus

## Competing interests

The author declares that he has no competing interests.
